# Candidate Key Proteins in Tinnitus—A Bioinformatic Study of Synaptic Transmission in the Cochlear Nucleus

**DOI:** 10.3390/biomedicines12071615

**Published:** 2024-07-19

**Authors:** Johann Gross, Marlies Knipper, Birgit Mazurek

**Affiliations:** 1 Tinnitus Center, Charité-Universitätsmedizin Berlin, 10117 Berlin, Germany; birgit.mazurek@charite.de; 2 Leibniz Society of Science Berlin, 10117 Berlin, Germany; marlies.knipper@uni-tuebingen.de; 3 Department of Otolaryngology, Head and Neck Surgery, Tübingen Hearing Research Center (THRC), Molecular Physiology of Hearing, University of Tübingen, 72076 Tübingen, Germany

**Keywords:** auditory perception, acoustic stimulation, cochlear nucleus, synaptic transmission, tinnitus

## Abstract

The aim of this study was to identify key proteins of synaptic transmission in the cochlear nucleus (CN) that are involved in normal hearing, acoustic stimulation, and tinnitus. A gene list was compiled from the GeneCards database using the keywords “synaptic transmission” AND “tinnitus” AND “cochlear nucleus” (Tin). For comparison, two gene lists with the keywords “auditory perception” (AP) AND “acoustic stimulation” (AcouStim) were built. The STRING protein–protein interaction (PPI) network and the Cytoscape data analyzer were used to identify the top two high-degree proteins (HDPs) and their high-score interaction proteins (HSIPs), together referred to as key proteins. The top1 key proteins of the Tin-process were BDNF, NTRK1, NTRK3, and NTF3; the top2 key proteins are FOS, JUN, CREB1, EGR1, MAPK1, and MAPK3. Highly significant GO terms in CN in tinnitus were “RNA polymerase II transcription factor complex”, “late endosome”, cellular response to cadmium ion”, “cellular response to reactive oxygen species”, and “nerve growth factor signaling pathway”, indicating changes in vesicle and cell homeostasis. In contrast to the spiral ganglion, where important changes in tinnitus are characterized by processes at the level of cells, important biological changes in the CN take place at the level of synapses and transcription.

## 1. Introduction

About 10–15% of people worldwide suffer from tinnitus. They hear sounds that are objectively not present. Tinnitus is a pathological activity of the CNS that has a high morbidity value for many of the afflicted and requires treatment [1].

Perception of sounds is linked to a functioning cochlea with its spiral ganglion and subsequent auditory centers in the brainstem and cortex. The first center in the brainstem is the cochlear nucleus. This center receives acoustically induced signals from the afferent fibers of the spiral ganglion and processes them for transmission to higher auditory centers in the CNS [2,3]. In addition, signals from somatosensory, vestibular, and higher auditory centers are processed [4]. The CN complex consists of at least three subnuclei: the anteroventral cochlear nucleus (AVCN), the posteroventral cochlear nucleus (PVCN), and the dorsal cochlear nucleus (DCN). After experimental removal of the cochlea, degeneration occurs in the CN in three phases: (1) acute degeneration, especially of nerve fibers; (2) a decrease in the number of neurons and incipient changes in synaptic plasticity; and (3) CN reorganization and neuroplastic changes [5].

All tinnitus theories posit that the causes of tinnitus (e.g., noise, salicylate) induce remodeling of synaptic transmission in the cochlear nucleus (CN) and other nuclei of the auditory pathways and that these nuclei could thus become centers contributing to tinnitus development [6,7,8]. This is supported by the observation that tinnitus persists even after auditory-nerve transection [7]. Noise, deafferentation, and synaptopathy are associated with hyperexcitability in the CN and other auditory nuclei. There is agreement that noise, deafferentation or synaptopathy is necessary for the development of tinnitus, but not sufficient [8,9,10,11]. Remodeling processes identified in tinnitus are described by terms such as “central neural gain”, “homeostatic plasticity”, and “neural synchrony” and are interpreted as compensatory reactions. Changes in synaptic transmission due to causes of tinnitus include long-term excitatory/inhibitory imbalance, long-term depression, and long-term potentiation, which are important elements of neural gain and homeostatic regulatory mechanisms [12,13,14]. Ultimately, degeneration of nerve fibers, activation of neuroplastic changes and repair processes associated with fiber growth, and synaptogenesis induce a new balance of excitatory and inhibitory activity, resulting in tinnitus and hyperacusis [8,15].

These processes involve a large number of proteins that constantly interact with each other. Bioinformatics methods have become a powerful tool for analyzing key proteins of biological structures or processes [16]. Databases that assign genes and proteins to specific symptoms or diseases are fundamental for this approach [17]. The quantitative analysis of the interactions of these genes and proteins in a network enables the identification of key proteins for the respective biological process or disease. The comparison of the key proteins under physiological conditions and those of the disease enables insight to be gained about the mechanism of the disease and the identification of biomarkers.

Thus far, it has not been sufficiently investigated which proteins play a key role in tinnitus in the cochlear nucleus. It is of great interest to understand which molecular changes are detectable in the different auditory complexes and whether they are related to functional changes in these regions [18,19]. The methods of bioinformatics and the existence of extensive freely accessible databases increasingly allow the identification of proteins that play an important role in physiological and pathological biological processes characteristic of symptoms or diseases [20]. The aim of the present study is to identify key proteins in the CN and discuss their biological function in tinnitus. Understanding the molecular biological mechanisms can help in the development of appropriate therapies.

## 2. Materials and Methods

### 2.1. Approach

**Figure 1:**
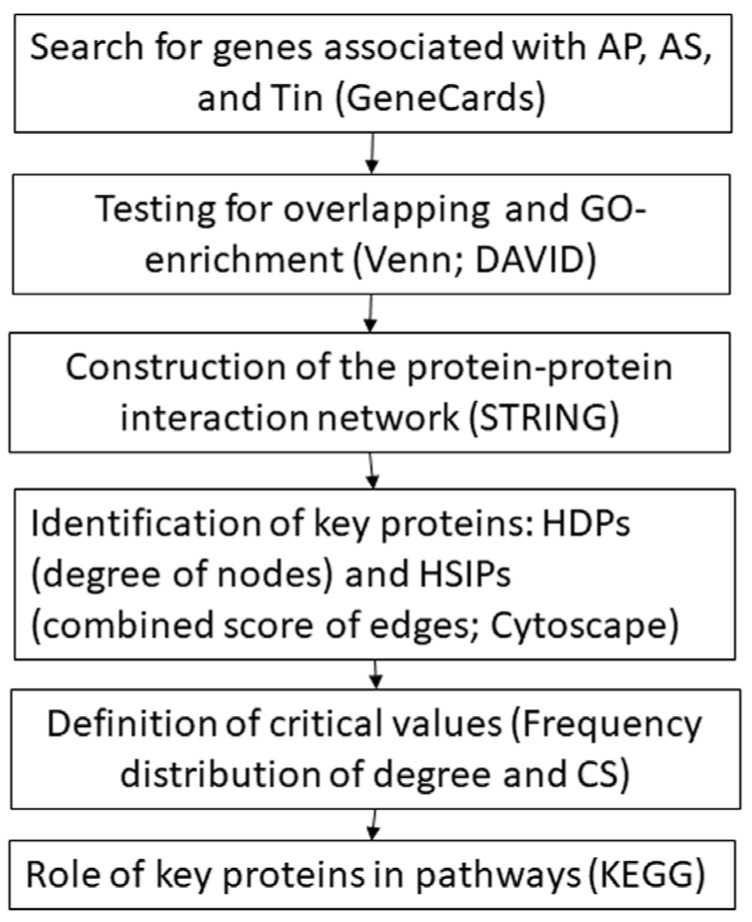
Workflow for studying the key proteins. AP—auditory perception; AS—acoustic stimulation; Tin—tinnitus; HDP—high-degree protein; HSIP—high-score interaction protein; CS—combined score.

Synaptic transmission and synaptic plasticity are fundamental biological processes of normal hearing and of tinnitus. The flow diagram illustrates our approach to assess differences in synaptic transmission between normal perception of sounds and tinnitus at the molecular level (Figure 1). Initially, we searched for three gene lists from the GeneCards database (https://www.genecards.org/ accessed on 6 April 2024 [17]) using the following keywords: (a.) “Cochlear nucleus” AND “synaptic transmission” AND “auditory perception” (including the terms “perception of sound” AND “normal hearing”), indicative of the physiological hearing process and summarized under the term “Auditory Perception” (AP); (b.) “Cochlear nucleus” AND “synaptic transmission” AND “acoustic stimulation” (AcouStim), indicative of activation of the auditory system (AcouStim); and (c.) “Cochlear nucleus” AND “synaptic transmission” AND “tinnitus” (Tin).

### 2.2. Characterization of the Gene Lists

The gene lists were characterized by analyses of gene overlap using Venn diagrams (http://bioinformatics.psb.ugent.be/webtools/Venn/ accessed on 17 May 2024). For the gene-enrichment analysis and the identification of Gene Ontology (GO) terms, the Database for Annotation, Visualization, and Integrated Discovery (DAVID) was used (https://david.ncifcrf.gov/ accessed on 17 May 2024; [21]).

### 2.3. Analysis of the Protein–Protein Interaction

The construction of the protein–protein interaction (PPI) network was performed using the STRING database (Search Tool for the Retrieval of Interacting Genes; https://string-db.org/ accessed on 21 May 2024; [22]). The Cytoscape data analyzer (https://cytoscape.org/ accessed on 29 May 2024) was used to identify functionally important proteins in the PPI network. As criteria for functionally important proteins, two types of criteria were used: (a) the number of degrees and (b) the combined score (CS) for interacting proteins. In addition to the degree, the betweenness centrality (“importance” of the node in the network) and the closeness centrality (a measure of how fast information spreads from a given node to other reachable nodes in the network) were considered. Because of different biases within the list of proteins, and to limit the study, only the top two high-degree proteins (HDPs) were selected for analysis. The CS of proteins interacting with HDPs includes, among other data, coexpression, the experimentally determined interactions, and automated text mining [20]. We have hypothesized that the HDPs and the corresponding high-score interaction proteins (HSIPs), named key proteins, play a functionally important role in the regulation of protein–protein networks. In this study, as the critical value for HSIPs, we chose proteins with a CS value > 90th percentile of the frequency distribution curve.

### 2.4. Pathways and Synaptic Proteins

The KEGG database was used to identify the molecular pathways in which key proteins and their HSIPs act together (https://www.genome.jp/kegg/pathway.html/ accessed on 4 June 2024). The following databases served for brief definition and characterization of proteins or genes: https://www.uniprot.org/uniprotkb/; https://syngoportal.org; https://thebiogrid.org/tt//cpdb.molgen.mpg.de/; https://www.ncbi.nlm.nih.gov/ accessed between 4 April 2024 and 4 June 2024.

## 3. Results

### 3.1. Characterization of the Gene Lists

**Figure 2:**
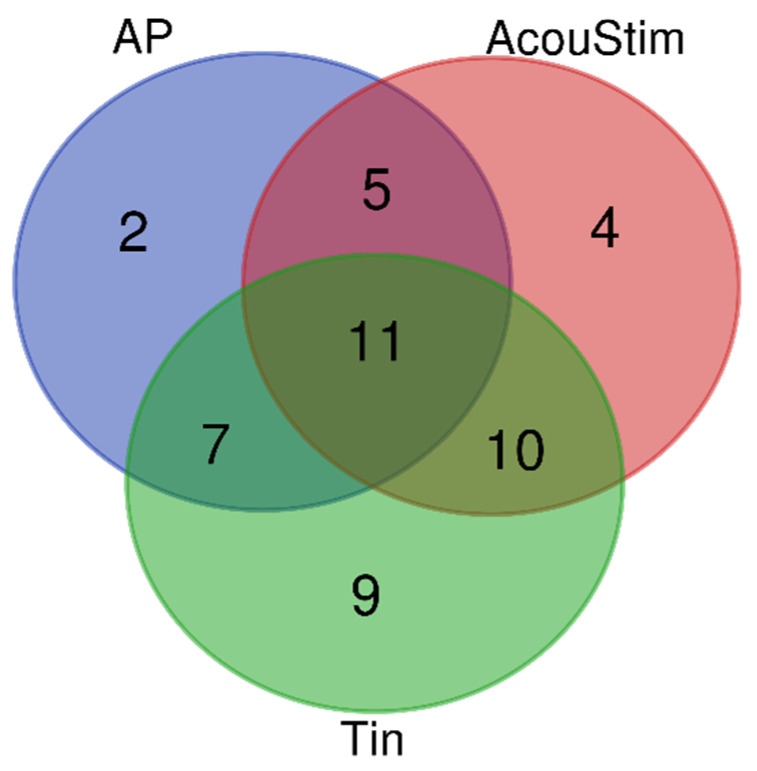
Venn diagram. AP—auditory perception, AcouStim—acoustic stimulation, Tin—tinnitus.

The gene lists for AP, AcouStim, and Tin differed in both the number and type of genes (Appendix A Table A1, Table A2 and Table A3). For the AP group, 25 genes were identified; for the AcouStim group, 30 genes; and for the Tin group, 37 genes. As an indicator of the relevance of each gene to the respective process, the numerical value of the score ranged from 0.95 to 21.2. To characterize the three gene lists, analysis of overlapping (Venn diagram) and GO enrichment analysis were used. The overall number of unique genes was 48, of which 11 genes were associated with the AP, AcouStim, and Tin processes; nine genes were associated with Tin process (Figure 2 and Table 1). There was a significant overlap among genes associated with the AP, AcouStim, and Tin processes.

The GO enrichment analysis indicated 42 chart records for the AP group, 100 chart records for the AcouStim group, and 218 records for the Tin group (*p* < 0.01). To limit the volume of the study, only the top five GO terms with the highest significance for cellular components (CC) and for biological processes (BP) were selected from the DAVID database (Table 2). The top five GO terms under cellular components and under biological processes for the AP and AcouStim gene lists reflected neural structures and functions known for normal auditory processing (e.g., chemical synaptic transmission, excitatory postsynaptic potential), but also for more specific processes in the auditory system. In the AcouStim process, the GO terms “regulation of postsynaptic cytosolic calcium ion concentration” and “response to xenobiotic stimulus” may indicate processes of activation of the CN neuronal complex. In the Tin process, GO terms such as “nerve growth factor signaling”, “nervous system development”, and “negative regulation of neuron apoptotic process” may indicate processes of reorganization.

### 3.2. PPI Network of the AP, AcouStim, and Tin Processes

**Figure 3:**
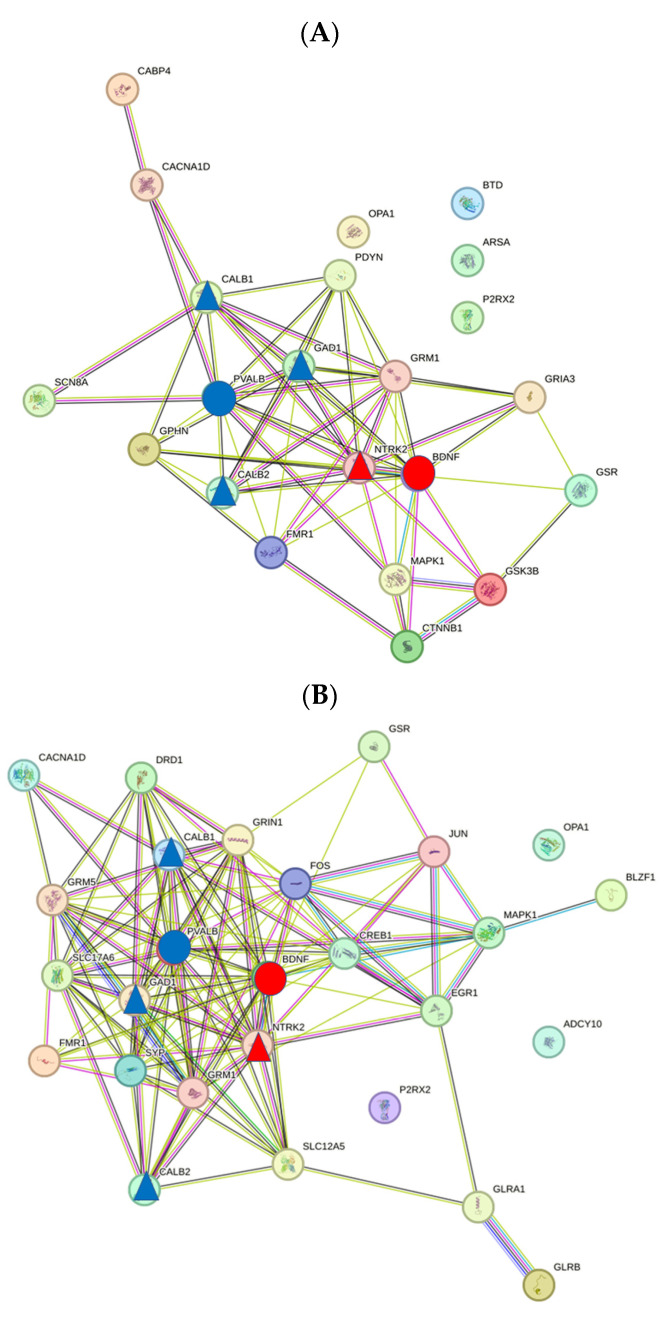

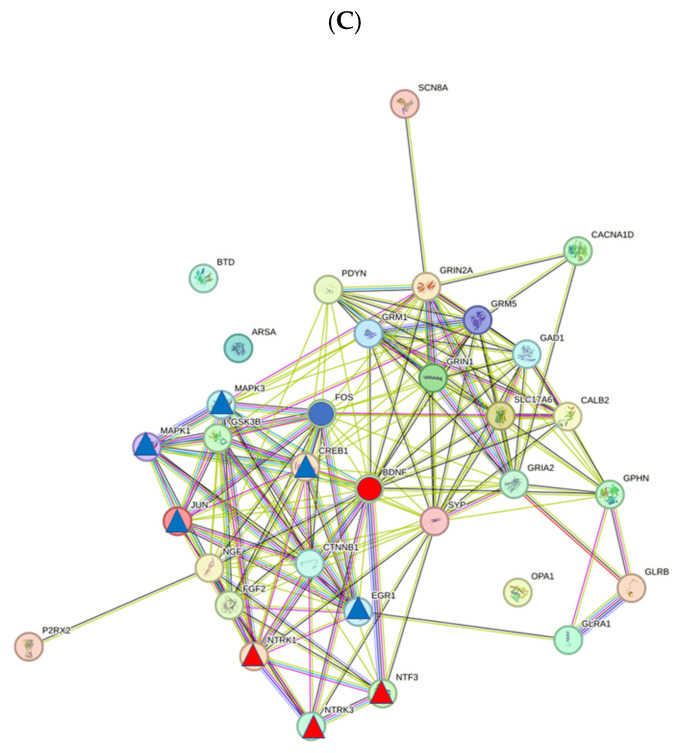
PPI network of the AP, AcouStim, and Tin processes in the CN. (**A**) AP; (**B**) AcouStim; (**C**) Tin. Top HDP—red circle; Top2 HDP—blue circle. Triangles—HSIPs. Topological criteria (AP/AcouStim/Tin): number of nodes—18/24/30; number of edges—62/122/176; avg. number of neighbors—7.0/10.2/11.73; network (NW) radius (2/2/2); characteristic path length—1.77/1.74/1.71; NW heterogeneity—0.52/0.50/0.47; NW centralization 0.47/0.42/0.45.

When comparing the gene lists with the PPI network, it was noticeable that the number of proteins for each network was smaller than the number of genes (Table 1). This is caused by the presence of genes in the list that only encode transcripts; for example, BDNF-AS, with a very high tinnitus score (18.2), exerts its effects via the regulatory mechanism of microRNA [23]. Proteins involved in specific forms of hearing disorders as a result of mutations (e.g., P2RX2) are also not part of the PPI network [24]. The topological parameters show only small differences between the three networks (legend of Figure 3). However, the k-mean analysis of STRING pointed to two clusters in the Tin network. Cluster 1 contained mainly proteins of glutamatergic synapses, and cluster 2 contained mainly proteins of the neurotrophin signaling pathway. It was noticeable that all key proteins were localized in cluster 2 (Figure 3; see paragraph on key proteins).

**Figure 4:**
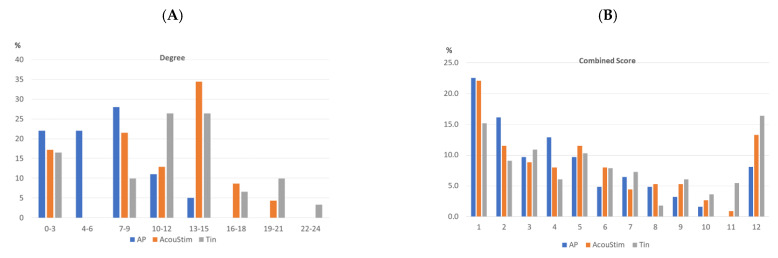
Frequency distribution of the degree (**A**) and CS values (**B**) of the AP, AcouStim, and Tin networks. The frequency distribution of the degree and CS values was calculated as the percentages of the number of nodes and edges (See legend of Figure 3). The intervals of degree values are 2, and those of CS values are 50. The CS values of class 1 correspond to CS = 400–450, and those of class 12 correspond to CS = 950–999. Characteristic values of the degree frequency curves (AP/AcouStim/Tin): Median—7/12/12; 90th percentile—12/17/19. Characteristic values of the CS frequency distribution: Median—555/598/647; 90th percentile—865/966/991.

In addition to the topological parameters, we characterized the networks by analyzing the frequency distribution of the degree and CS values. The frequency of the degree values followed a bell-shaped histogram (Figure 4A), whereby the frequency of the degree values of the Tin group shifted to higher values compared with the AP and AcouStim groups. The frequency of the CS values decreased with increasing CS values for all groups up to CS values of about 900 (Figure 4B). The frequency of CS values > 950 in the Tin group was nearly twice that of the AP group. Similar frequency curves were found in the spiral ganglion [25]. The 90th percentile of the frequency distribution curve was used as the critical CS value for identification of HSIPs (AP/AcouStim/Tin—865/966/991; Table 3).

### 3.3. Key Proteins of the PPI Networks of the AcouStim and Tin Lists

The top two HDPs of each group and the corresponding HSIPs were selected as key proteins (Table 3). It was noticeable that the key proteins of AP and AcouStim were practically identical. In the AP and AcouStim networks, BDNF and PVALB were the proteins with the highest degree values. In the AP and AcouStim processes, the top protein, BDNF showed close interactions with NTRK2, and the second-tier protein, PVALB, interacted closely with GAD1, CALB1, and CALB2. In the Tin group, the top protein, BDNF, and top2 protein, FOS, showed close interactions with more proteins: BDNF with NTRK3, NTRK1, and NTF3 and FOS with JUN, CREB1, EGR1, MAPK3, and MAPK1.

GO enrichment analysis of the key proteins showed clear differences between the AP/AcouStim processes on the one hand and the Tin process on the other (Table 4). With regard to the cellular components, the key proteins of the AP/AcouStim processes were assigned to structures of the synapse (e.g., axons, dendrites). The key proteins of the Tin process were also assigned to axons, but in addition to transcription and regulation of endo- and exocytosis of vesicles (“late endosome”, “endoplasmic reticulum lumen”). The CC term “late endosome” points to the role of endocytosis and endosome retrieval mechanisms in the generation of synaptic vesicles [26,27]. With regard to biological processes, the proteins of the AP/AcouStim process were associated with the BDNF signaling pathway and the regulation of synaptic transmission. In contrast to the AP/AcouStim process, the key proteins of the Tin process indicated processes such as “cellular response to cadmium ions”, “cellular response to reactive oxygen species”, and “nerve growth factor signaling pathway (Table 4). The GO process “cellular response to cadmium ions” is not to be taken literally, but rather refers to biological processes that play a role in heavy metal intoxication, such as changes in ion homeostasis or in cell division, and the initiation of apoptosis and necrosis [28].

### 3.4. Proteins Interacting with BDNF in the AP, AcouStim, and Tin Processes

**Figure 5:**
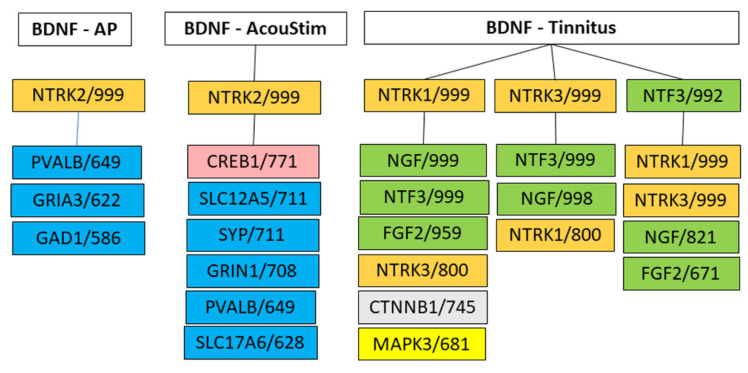
Interactions of BDNF and its HSIPs with proteins characterized by a CS value > median. Meanings of colors: orange—members of the neurotrophic tyrosine receptor kinase (NTRK) family; pink—transcription factor; blue—proteins directly involved in synaptic transmission; green—growth factors; yellow—members of the MAP kinase family (alias extracellular signal-regulated kinases, ERKs), light gray—CTNNB1. Numbers indicate the corresponding CS values.

To characterize the role of the tinnitus-related top1 HDP, BDNF, in the AP, AcouStim, and Tin processes, and to identify pathways of BDNF, we searched for proteins that showed interactions with HSIPs of BDNF and thus extended the number of proteins. As a criterion for interactions of the HSIPs with other proteins, we used the value of CS >median of the frequency distribution curve (AP CS > 555, AcouStim CS > 598; Tin CS > 647). This analysis showed remarkable differences between the AP, AcouStim, and Tin processes (Figure 5). Despite the fact that the AP and AcouStim processes had identical key proteins, the proteins with which NTRK2 interacted differed significantly. In the AP process, BDNF interacted with NTRK2, PVALB, GRIA3, and GAD1. In the AcouStim process, BDNF also interacted with NTRK2, but NTRK2 interacted with several synaptic proteins. In the Tin process, BDNF interacted with NTRK1, NTRK3, and NTF3; these proteins interacted with the growth factors NGF, NTF3, and FGF2 as well as CTNNB1. Thus, NTRK2 had clearly different interaction proteins than NTRK1 or NTRK3: typical interaction proteins for NTRK2 were PVALB, GAD1, and synaptic proteins, whereas important interaction proteins for NTRK1 and NTRK3 were growth factors.

### 3.5. Pathways

Based on the proteins that closely interact with HDP and HSIP (Figure 5), six KEGG pathways were identified that contain BDNF as a member and that may play an important role in synaptic transmission in the AP, AcouStim, and Tin processes (Table 5). The following pathways showed the highest significance: (1) The neurotrophin signaling pathway (hsa04722) plays an important role in signal transduction of nerve growth factors, in neural development, and in interactions with several pathways (e.g., MAPK and PI3K signal pathways). (2) Alcoholism (hsa05034) appears as a pathway in the list because BDNF, CREB1, GRIN1, and NTRK2 are proteins that are also changed in this chronic disorder [29]. (3) The cAMP signaling pathway (hsa04024) plays an important role in metabolism, calcium homeostasis, cell fate, and gene transcription. (4) The Ras signaling pathway (hsa04014) is involved in the switching of several signaling pathways regulating multiple cellular functions (survival, proliferation, migration, growth, and differentiation). (5) The MAPK signaling pathway (hsa04010) is a highly conserved pathway that is involved in cell proliferation, differentiation, and migration. (6) The phosphatidylinositol 3′-kinase (PI3K-Akt; hsa04151) signaling pathway regulates cellular functions such as transcription, translation, proliferation, growth, and survival. Remarkably, the growth factors NGF, NTF3, and FGF2 are only present in the pathways associated with tinnitus.

## 4. Discussion

A comparison of the candidate key proteins in the CN of AP and AcouStim processes with those in Tin revealed similarities and differences. The top HDP candidate key protein for all three processes was BDNF. However, there were clear differences in the interaction of BDNF with other proteins, as measured by CS. The top2 HDPs differed between AP/AcouStim and Tin, both in the protein itself and in the HSIPs.

### 4.1. Key Proteins in the AP and AcouStim Process

*Top1—BDNF-NTRK2:* BDNF is involved in all elementary regulatory processes such as survival and differentiation of neurons, axonal growth, dendrite growth, migration, path finding, transcription, translation, transport, secretion, maturation, differentiation, growth, regeneration or migration, synaptic transmission, and plasticity [30,31]. BDNF and NTRK2 were also identified as key proteins in the AP/AcouStim process in the spiral ganglion [25]. BDNF modulates its own mRNA expression via activation of TrkB receptors and the MAPK signaling pathway [32].

NTRK2 (Neurotrophic Receptor Tyrosine Kinase 2; also known as BDNF/NT-3 Growth Factors Receptor, TRKB-Tropomyosin-Related Kinase B) is a membrane receptor. It is a member of the TRK family that in addition consists of NTRK1 and NTRK3 (TRKA, TRKC). The affinity for binding of the different neurotrophins to the kinase receptors differs; BDNF binds and activates mainly NTRK2; in the case of NGF, NTRK1; and in the case of NT3, NTRK3. NT3 also binds NTRK2 and NTRK1 with weaker affinity [33,34]. Activation of the receptors by neurotrophins, specifically by BDNF, promotes neuronal survival, neuronal differentiation, axon and neurite growth, and synaptic transmission and plasticity. Binding of BDNF alters the structure of the receptor (dimerization, mutual phosphorylation), the receptor thus binds a number of adaptor and signaling proteins and via the transcription factor CREB activates, among others, protein kinase B (AKT), and mitogen-activated protein kinase (MAPK) [35].

Top2—PVALB-GAD1-CALB1-CALB2: PVALB (parvalbumin) is a Ca^++^ binding protein that is detectable in axons, dendrites, and synapses. Depending on their activity, it is expressed in neurons, especially in GABAergic inhibitory interneurons (PV+ interneurons; PV+-Ins). Since it has buffering properties, it regulates the local neuronal Ca^++^ concentration, and thus modulates the excitatory and inhibitory activities of the neurons. Simultaneously, it protects neurons from Ca^++^ overload [36]. Synapse formation between PV+-Ins and projecting neurons in the auditory path seems to be a precondition for development of tinnitus [37,38]. PV+-Ins are inhibitory cells that play a fundamental role in maintaining the balance of excitation and inhibition throughout the CNS [37,39]. The inhibitory drive of PV+-Ins can be influenced by input, output, and intrinsic properties of the cells. The cells are fast-spiking and form afferent and efferent connections. Their function is linked to a high metabolic demand, which increases the vulnerability of the cells, in, e.g., ischemic conditions.

GAD1 (GAD-67) is an enzyme that catalyzes the formation of the inhibitory neurotransmitter GABA from glutamic acid. The regulation of expression occurs via BDNF-dependent and BDNF-independent signaling pathways, as well as via neuronal activity. Activity-dependent regulation of GAD1 expression is controlled by Ca^++^-dependent mechanisms [40]. The association of GAD1, GABA, and CALB1 are important for the balance of excitatory and inhibitory activity in the DCN. The expression of GAD1 and CALB1 changes over time, with temporal fluctuations, suggesting that there is a permanent, input-dependent adjustment of excitatory and inhibitory activity in the CN [41]. Reduced expression of GAD1 leads to a reduced level of GABA, and thus to reduced inhibition [8].

CALB1 (Calbindin1; also known as CALB; D-28K) and CALB2 (Calretinin) belong to the group of calcium-binding proteins such as calmodulin and troponin C. Under physiological conditions, Ca^++^ is not a free ion in the cytoplasm, but it is bound to proteins that act as Ca^++^ buffers [42] and protectors against toxic concentrations of Ca^++^, thereby regulating important cellular processes. CALB1 is expressed in glial cells, but also in neuronal cells [43] and is involved in the regulation of pre- and postsynaptic cytosolic calcium levels. The expression of CALB1, CALB 2, and parvalbumin decreases in ventral CN during aging, and may contribute to hearing deterioration in old age [44]. Korada and Schwartz [45] observed a possible relationship between parvalbumin, calbindin and glutamate receptors in octopus cells of CN.

In summary, the normal (AP) and activated status (AcouStim) in CN are characterized by the BDNF receptor signaling pathway and by NTRK2 as the receptor. This pathway is associated with the biological processes “regulation of long-term synaptic potentiation” and “regulation of presynaptic cytosolic calcium ion concentration”. The fine regulation of BDNF-NTRK2 signaling occurs via the regulation of GAD1 activity and Ca^++^ levels by the top2 key proteins PVALB, CALB1, and CALB2.

### 4.2. Key Proteins in the Tin Process

*Top1—BDNF-NTRK1-NTRK3-NTF3*: In the Tin process, BDNF also appears as the top1 HDP, and it interacts closely with NTRK1, NTRK3, and NTF3 at a similar level. The associated HSIPs differ significantly compared to the AP/AcouStim process: In AP/AcouStim, the most important HSIP is NTRK2; in Tin, NTRK1, NTRK3, and NTF3 are the most important HSIPs. All three TRK receptors (NTRK1/TrkA, NTRK2/TrkB, and NTRK3/TrkC) are expressed in the CN [46] with differences detectable in different cells. Small round neurons and fusiform cells express TRKA and TRKB, and multipolar, large, and octopus cells appear to express all three receptors. TRKA and TRKB immunoreactive cells appear to have inhibitory properties, whereas neurons that also express TRKC exhibit excitatory properties.

NTRK1 (neurotrophic receptor tyrosine kinase 1) is a high-affinity NGF receptor involved in the regulation of proliferation, differentiation, and the survival of neurons. NTRK1 can also bind and be activated by NTF3 (neurotrophin-3). NTF3 supports axonal extension through NTRK1, but has no effect on neuron survival. In a model using traumatic noise, Manohar et al. [7] found an increase in NTRK1 expression on day 28 after trauma (126 dB SPL, hearing loss 50–60 dB). Activation of NTRK1 is thought to be important for neurite formation and reorganization of neuroplasticity. NGF is activated in damaged and inflammatory tissues, and the binding of NGF to NTRK1 results in decreased inflammation. It is generally assumed that the activation of NTRK3 leads to similar results as the activation of NTRK2 by BDNF, including synaptic plasticity [47]. NTRK3 is thought to play a role in the changes in synaptic activity associated with childhood-onset mood disorders [48]. The target proteins of the various NTRKs have, however, not yet been sufficiently investigated.

NTF3 (neurotrophin 3, also known as NT3) belongs to the same group of neurotrophins as BDNF and NGF. NTF3 binds to the NTRK3 receptor and regulates not only the survival of neuronal cells via this signaling pathway but also their differentiation and synaptogenesis [49,50]. In cortical neurons, NTF3 also influences spontaneous activity, synchronization of excitatory activity, and inhibitory synaptic transmission via GABA receptors [49]. After unilateral hearing loss, microglial cells are activated and NTF3 is activated in neurons, but also in glial cells [50]. BDNF and NTF3 have different effects on biological processes in a cell culture of CN (mouse): BDNF stimulates cell survival and axon growth, NTF3 only has a strong influence on neuronal survival [51]. After unilateral cochlear ablation in guinea pigs, there are significant changes in NTF3 and BDNF levels [52]. In combination with NGF, BDNF has a strong influence on neurite growth [53].

*Top2—FOS-JUN-CREB1-EGR1-MAPK3-MAPK1:* The top2 HDP of the Tin process is FOS; it shows HSIs with the transcription factors JUN, CREB1, and EGR1, as well as the signaling molecules MAPK1 and MAPK3 at a similar significance level. FOS, JUN, EGR1, and CREB1 are expressed in the CNS and play a role in CNS development, as well as in damage (neurodegeneration, hypoxia–ischemia) and long-term potentiation [54]

FOS is a transcription factor that can be activated by stimuli such as c-AMP, membrane depolarization, excitatory neurotransmitters, elevated Ca^++^ levels, and growth factors (NGF). c-Fos belongs to the group of immediate-early genes (IEGs) and shows close interactions with the transcription factors JUN, CREB1, and EGR1 [55]. IEGs are important regulators of growth and differentiation that respond very sensitively to corresponding signals [56]. The intensity and duration of acoustic stimulation of young rats influences the expression of c-fos and JunB in the CN and inferior colliculus. Traumatic acoustic stimuli can lead to hearing loss and tinnitus; these processes are associated with changes in BDNF and c-Fos expression [57]. c-Fos is considered to be an indicator of neuronal activity [58,59].

CREB1 (cAMP responsive element binding protein 1, also known as CREB) is a transcription factor and DNA-binding protein. CREB1 is a significant factor for neuronal survival, neuronal migration, synaptogenesis, and long-term potentiation (LTP; [32]). BDNF is an important target gene of CREB [60]. It is assumed that BDNF, NTRK2, and CREB form an autoregulatory loop [32]. The transcription factor CREB1 plays an important role in the expression of IEG and NGF, and is thus a critical mediator for long-term adaptation to incoming stimuli and synaptic plasticity [56].

MAPK3 (also known as extracellular signal-regulated kinase 1, ERK1) and MAPK1 (ERK2) belong to the family of mitogen-activated protein kinases or extracellular signal-regulated kinases that are involved in the regulation of numerous cellular processes in the brain. Such processes include synaptic plasticity, brain development, neuroinflammation, neuronal cell death, learning and memory, neurodegeneration, regulation of transcription factors, and transcriptional activity [61,62]. The mitogen-activated protein kinase MAPK3 (ERK1) can be activated by neurotrophins, neuronal activity, or cAMP and influences the development, survival, and differentiation of neuronal cells, including long-term potentiation [63]. In the CN, the MAP signaling pathways play a major role in adaptive changes after deafferentation [64]. The close associations of BDNF and NGF in the Tin network are likely mediated by MAPKs [65]. The multiple functions of MAPK can be found in the excellent review of [62].

In summary, top1 key proteins in the Tin process reflect biological processes such as “cellular response to cadmium ion”, “cellular response to reactive oxygen species”, “nervous system development”, indicating processes of modulation of synaptic transmission [66]. The top2 key proteins indicate that the mechanisms of regulation of these processes occur on the level of transcription. The dominate role of transcription factors is an indication of changes in neuronal activity; conversely, it can be assumed that a change in transcription induces changes in neuronal activity [67].

### 4.3. GO Enrichment Analysis of Key Proteins

A comparison of the GO terms based on the complete gene lists (Table 2) to those verified on the basis of the key proteins (Table 4) shows good agreement for the AP and AcouStim processes for both the CC and BP terms. The good agreement is an indication that the identified key proteins are indeed important proteins of the respective biological process. For the tinnitus process, the GO terms based on the key proteins appear to be much more specific. The GO enrichment analysis (CC) of the key proteins of the Tin process indicates changes in the formation and use of synaptic vesicles, indicated by the GO term “late endosome”, and by the different transcriptional complexes (Table 4). The continuous availability of synaptic vesicles is of fundamental importance for synaptic transmission. This is made possible by the efficient local supply of vesicle membranes, the recycling, and the regulated transport of these proteins [68]. The regulation of early and late endosomes is of great importance for long-term potentiation (LTP) and long-term depression (LTD; [69]) which play an important role in tinnitus [70,71]. GO enrichment analysis (BP) of key proteins indicates changes that show similarities to those of the cellular response of neurons to heavy metals or ROS [72,73] inducing pathological changes in synaptic plasticity in the CN [71]. Under the influence of the altered synaptic signals from the spiral ganglion and the altered somatosensory and vestibular inputs due to noise and auditory disturbances, the LTP and LTD in the CN change [74]. In a salicylate-induced Tin model, structural changes in synaptic vesicles associated with changes in the expression of *Grin2b* and *Egr-1* were detected in the DCN [75].

### 4.4. Synaptic Transmission in the AcouStim and Tin Processes

**Figure 6:**
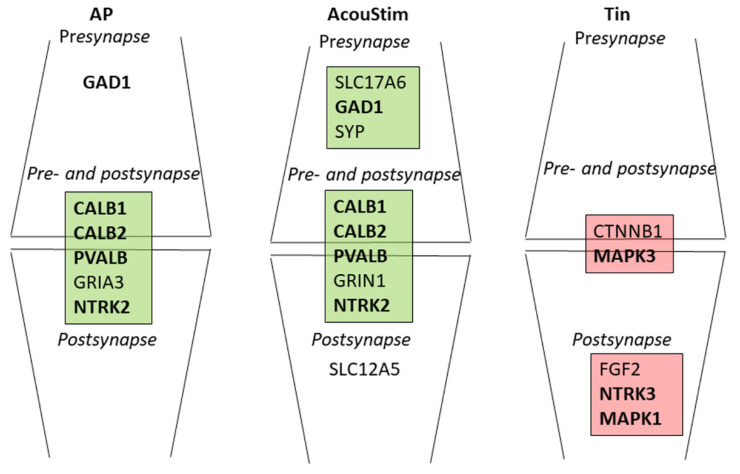
Synaptic locations and biological functions of key proteins and proteins with close interaction with BDNF according to the SynGO database. Bold letters are key proteins.

To better understand the specificity of the synaptic transmission of the AP, AcouStim, and Tin processes, we compared the localization and biological function of key proteins (Table 3) and the proteins that are closely associated with BDNF (Figure 5), based on the SynGO database (https://syngoportal.org/ accessed on 4 June 2024 [76]; Figure 6).

In the AP and AcouStim processes, NTRK2 (transsynaptic signaling); GAD1 (GABA synthesis); and the calcium -binding proteins CALB1, CALB2, and PVALB (calcium signaling) play an important role. However, there are also differences: in the AP process (normal hearing), the GRIA3 receptor (transmitter-gated ion channel activity involved in regulation of pre- and postsynaptic membrane potential) appears in addition, as do, in the AcouStim process (stimulated activity), SLC17A6 (vesicle neurotransmitter loading), SYP (modulation of synaptic transmission), GRIN1 (regulation of ion channel activity), and SLC12A5 (regulation of postsynaptic assembly).

In the Tin process, CTNNB1 and MAPK3 appear on the pre- and post-synaptic sides, and FGF2, NTRK3, and MAPK1 on the post-synaptic side. The role of these proteins requires special emphasis. *CTNNB1* (beta-catenin) is a protein of great importance for protein–protein contact, regulation of transcription, synaptic structural organization, and synaptogenesis [77]. By binding to cadherin and other proteins, beta-catenin acts as a scaffolding protein for the arrangement of synaptic vesicles in the presynaptic active zone and is thus involved in the localization and transport of vesicles [78]. *MAPK3* and *MAPK1* are important signaling molecules in synaptic plasticity and memory and are involved in the coordination of extracellular signals in neurons [79,80]. They participate in synaptogenesis inside the ventral cochlear nucleus [64]. *FGF2* is released from astrocytes and, via effects on Na^+^ currents, increases excitability and speed of transmission in neurons [81]. FGF2 is also involved in glutamate release in cerebral cortical neurons and interacts thereby with members of the MAPK pathway [82]. *NTRK3* is important for neurotrophin signaling, the regulation of synapse assembly, and synaptic plasticity; it also contributes to mood disorders [48].

### 4.5. Conclusions

The comparison of the candidate key proteins for synaptic transmission under physiological conditions and the awareness of sounds in tinnitus may provide insights into the underlying molecular mechanisms of tinnitus. However, the limitations of bioinformatic approaches should be noted, as discussed in a previous paper [25]. There are also further limitations. The CN is treated here as a “uniform structure”. In reality, the CN consists of several parts with different cellular features and differing neural activity [38,83,84].

Key proteins are expressions of dominant and global processes rather than processes of single cells, single axons, or dendrites. In the present study, we identified BDNF/NTRK2 and PVALB/GAD1/CALB as top 1 and top2 key proteins in the CN. The key role of BDNF in synaptic transmission in the auditory system and the role of PVALB/GAD1/CALB in excitatory/inhibitory balance are well established [8,18,30,31,38]. In tinnitus, the predominant activity is that of other proteins, namely, the top key proteins BDNF/NTRK3/NTRK1/NTF3 in interaction with a number of Top2 key proteins, all members of the IEGs. This leads to modulation of synaptic transmission and disruption of the balance between excitation and inhibition. The altered synaptic transmission is reflected in the absence of PVALB in the Tin gene list and an altered composition of synaptic proteins (Figure 6). Parvalbumin interneurons play a fundamental role in numerous neurological diseases, with a high probability of activity-regulated disinhibition or degeneration of these cells in the CN and thus the inhibitory function of these cells [85]. Parvalbumin interneurons are also discussed as key players in the context of the manifestation of tinnitus [86].

The key proteins NTRK3, NTRK1, and NTF3 in tinnitus are not simply involved in activating or inhibiting processes but in remodeling of synaptic transmission processes. This remodeling is expressed in the GO terms “late endosome”, “cellular response to cadmium” and “cellular response to reactive oxygen species” (Table 4). The top2 key proteins indicate that transcription is the most important molecular process for remodeling. In addition, the expression of immediate-early genes (IEGs) has been used as an indicator of neurons that undergo plastic changes [85]. The in-depth analysis of the targets of NTRK3/NTRK1/NTF3 under physiological and tinnitus conditions could help to create new starting points for therapeutic considerations.

PVALB is not detectable in the Tin gene list, and using degree and combined score values as indicators, the roles of CALB1, CALB2, and GAD1 are of minor importance in the Tin network. We assume that subsequently synaptopathy due to cochlear damage and the absence or alteration of high-spontaneous-rate signals in the auditory nerve are the cause of the reduction in the activity of PV+ interneurons, as discussed [37,86]. Thus, the present findings are consistent with theories of tinnitus and degeneration observed in the CN and define key proteins involved in this process. Key proteins could be used as markers for cellular and molecular changes in synaptic transmission associated with tinnitus in the CN. In addition, it is important to identify the mechanisms of these changes in the regions where the tinnitus originates.

In the CN, important biological processes in tinnitus are organized at the level of transcription, in contrast to the situation in SGNs, where important changes in tinnitus are characterized by processes of cell death and regeneration [25]. The important key proteins are BDNF, NGF, and NGFR in the spiral ganglion, in contrast to BDNF and transcription factors in the CN. The dominant processes of transcription indicate that there is a continuous adaptation regulated by afferent and efferent signals. Thus, the reorganization of synaptic transmission in the CN is very dynamic and complex and can be influenced by changes in the afferent and the efferent signals. Therapy for tinnitus should involve the restoration of the afferent and efferent signals.

## Figures and Tables

**Table 1:** Distribution of genes of the three gene lists among the different groups of the Venn diagram. AP—auditory perception; AcouStim—acoustic stimulation; Tin—tinnitus ^1^.

Process	Number	Genes
AP/AcouStim/Tin	11	GAD1 MALAT1 BDNF CALB2 BDNF-AS* MAPK1 GRM1 CACNA1D EMSLR P2RX2* OPA1*
AP/AcouStim	5	NTRK2 PVALB GSR CALB1 FMR1
AP/Tin	7	CTNNB1 GPHN GSK3B PDYN ARSA* SCN8A BTD*
AcouStim/Tin	10	SLC17A6 GLRB GLRA1 EGR1 GRIN1 FOS JUN GRM5 SYP CREB1
AP	2	CABP4 GRIA3
AcouStim	4	BLZF1 SLC12A5 ADCY10* DRD1
Tin	9	GRIA2 NTF3 GRIN2A PCAT1 NGF NTRK3 MAPK3 NTRK1 FGF2

^1^ The overall number of unique genes is 48. * Only in the gene list.

**Table 2:** Top five GO Terms for cellular components and biological processes in the CN for the AP, AcouStim, and Tin processes (*p* < 0.01).

AP (42 Chart Records)	AcouStim (100 Chart Records)	Tin (218 Chart Records)
*Cellular components: p = 3.5E-10 to 3.7E-5; fold enrichment 14–80*	*Cellular components: p = 1.3E-10 to 1.7E-6; fold enrichment 18–80*	*Cellular components: p = 7.8E-14 to 2.0E-5; fold enrichment 13–74*
-dendrite (10) -synapse (8) -axon (7) -terminal bouton (4) -axon terminus (4)	-dendrite (11) -neuron projection (10) -synapse (10) -terminal bouton (5) -dendritic spine (6)	-dendrite (14) -postsynaptic membrane (8) -neuron projection (8) -synaptic vesicle (6) -excitatory synapse (4)
*Biological processes: p = 1.1E-4 to 5.7E-3; fold enrichment 18–335*	*Biological processes: p = 4.4E-8 to 4.1E-4; fold enrichment 22–189*	*Biological processes: p = 9.1E-12 to 5.8E-6; fold enrichment 25–190*
-regulation of long-term synaptic potentiation (3) -chemical synaptic transmission (5) -brain-derived neurotrophic factor receptor signaling pathway (2)	-chemical synaptic transmission (8) -excitatory postsynaptic potential (4) -regulation of postsynaptic cytosolic calcium ion concentration (3) -response to xenobiotic stimulus (5) -chloride transmembrane transport (4)	-chemical synaptic transmission (11) -excitatory postsynaptic potential (6) -nerve growth factor signaling pathway (4) -nervous system development (8) -negative regulation of neuron apoptotic process (6)

In brackets: numbers of genes per GO term. Order of GO terms is determined by *p* values.

**Table 3:** Key proteins in the AP, AcouStim, and Tin networks.

HDP	Degree	Close	Betw	HSIP ^1^	Coexp	Exp	Text	CS	EB
*AP*									
BDNF	14	0.81	0.20	NTRK2	60	691	999	999	3.07
PVALB	12	0.77	0.21	GAD1	133	91	962	968	6.92
				CALB2	84	0	965	967	9.29
				CALB1	82	0	962	964	3.29
*AcouStim*									
BDNF	19	0.82	0.11	NTRK2	60	691	999	999	3.24
PVALB	17	0.77	0.07	GAD1	133	91	962	968	3.47
				CALB2	84	0	965	967	6.71
				(CALB1) ^2^	(82)	(0)	(962)	(964)	(4.46)
*Tinnitus*									
BDNF	24	0.85	0.11	NTRK3	65	65	999	999	17.5
				NTRK1	60	65	999	999	8.81
				NTF3	82	958	774	991	14.01
FOS	20	0.76	0.05	JUN	690	999	999	999	6.39
				CREB1	52	0	996	998	2.91
				EGR1	825	66	951	995	10.27
				MAPK3	55	450	907	994	4.79
				MAPK1	55	450	864	992	7.29

Abbreviations: Close—closeness centrality; Betw—betweenness centrality; HDP—high-degree protein; HSIP—high-score interaction protein; Coexp—coexpression, Exp—experimentally determined interaction; Text—automated text mining; CS—combined score; EB—edge betweenness. ^1^ CS > 90th percentile (AP—865, AcouStim—966, Tin—991); ^2^ slightly below the selected threshold value.

**Table 4:** GO processes of key proteins of the CN (*p* < 0.05).

AP/AcouStim (6 Proteins, 16 Charts)	Tin (10 Proteins, 170 Charts)
*Cellular components (proteins, fold enrichment); p = 4.7E-5 to 5.8E-3.*	*Cellular components (proteins, fold enrichment); p = 4.5E-4 to 4.4E-3.*
-terminal bouton (CALB1, CALB2, NTRK2; 282) -axon (BDNF, CALB1, NTRK2, PVALB; 39) -dendrite (BDNF, CALB1, CALB2, NTRK2; 31) -synapse (CALB1, CALB2, PVALB; 20)	-axon (BDNF, NTRK1, NTRK3, NTF3; 22) -RNA polymerase II transcription factor complex (3-FOS, JUN, CREB1; 50) -late endosome (MAPK1, MAPK3, NTRK1; 40) -transcription factor complex (FOS, JUN, CREB1; 26) -endoplasmic reticulum lumen (BDNF, MAPK1, MAPK3; 20)
*Biological processes (proteins, fold enrichment): p = 1.3E-3 to 3.9E-3.*	*Biological processes (proteins, fold enrichment): p = 4.8E-7 to 2.8E-5.*
-brain-derived neurotrophic factor receptor signaling pathway (BDNF, NTRK2; 1284) -regulation of long-term synaptic potentiation (CALB1, CALB2; 459) -regulation of presynaptic cytosolic calcium ion concentration (CALB1, CALB2; 428)	-cellular response to cadmium ion (FOS, JUN, MAPK1, MAPK3; 216) -cellular response to reactive oxygen species (FOS, JUN, MAPK1, MAPK3; 147) -nerve growth factor signaling pathway (BDNF, NTRK1, NTF3; 449) -nervous system development (FOS, BDNF, NTRK1, NTRK3, NTF3; 23) -transmembrane receptor protein tyrosine kinase signaling pathway (BDNF, NTRK1, NTRK3, NTF3; 57)

**Table 5:** Pathways involved in the AcouStim and Tin processes (*p* < 0.05).

Processes	Pathway	Members of the Pathway (HDP, HSIP)
AP	-Neurotrophin signaling	BDNF, NTRK2
	pathway (4.1E-2)	
AcouStim	-Alcoholism (1.9E-4)	BDNF, CREB1, GRIN1, NTRK2
	-cAMP signaling	BDNF, CREB1, FOS, GRIN1
	pathway (3.3E-4)	
	-Ras signaling pathway	BDNF, GRIN1, NTRK2
	(7.0E-4)	
Tin	-Neurotrophin signaling	BDNF, NGF, NTF3, NTRK1, NTRK3, MAPK3
	pathway (9.2E-9)	
	-Ras signaling pathway	BDNF, NGF, NTF3, FGF2, NTRK1, MAPK3
	(7.0E-4)	
	-MAPK signaling	BDNF, NGF, NTF3, FGF2, NTRK1, MAPK3
	pathway (9.7E-4)	
	-PI3K-Akt signaling	BDNF, NGF, NTF3, FGF2, NTRK1, MAPK3
	pathway (1.2E-3)	

Abbreviations and explanations: AP—auditory perception; AcouStim—acoustic stimulation; Tin—tinnitus. The table indicates only pathways that have BDNF as a member. In total, the AP process had only one KEGG pathway that reached the significance level of *p* < 0.05; the AcouStim process, 37 KEGG pathways; and the Tin process, 76 KEGG pathways.

## Data Availability

The original contributions presented in the study are included in the article.

## Appendix A. Gene Lists

**Table A1:** Genes identified by the keywords “cochlear nucleus” AND “synaptic transmission” AND “perception of sound” (PoS) OR “auditory perception” (AP) OR “normal hearing” (NH) ^1^.

Gene	Score	Name
PoS (n = 10)		
BDNF	13.59	Brain Derived Neurotrophic Factor
P2RX2	10.99	Purinergic Receptor P2X 2
GRM1	7.27	Glutamate Metabotropic Receptor 1
CTNNB1	4.63	Catenin Beta 1
MAPK1	4.55	Mitogen-Activated Protein Kinase 1
FMR1	3.52	Fragile X Messenger Ribonucleoprotein 1
BTD	3.33	Biotinidase
CACNA1D	3.04	Calcium Voltage-Gated Channel Subunit Alpha1 D
ARSA	1.94	Arylsulfatase A
OPA1	1.74	OPA1 Mitochondrial Dynamin Like GTPase
AP (n = 13)		
BDNF-AS	17.96	BDNF Antisense RNA
BDNF	14.64	Brain Derived Neurotrophic Factor
P2RX2	9.85	Purinergic Receptor P2X 2
MALAT1	7.64	Metastasis Associated Lung Adenocarcinoma Transcript 1
GRM1	7.52	Glutamate Metabotropic Receptor 1
NTRK2	6.70	Neurotrophic Receptor Tyrosine Kinase 2
PDYN	5.77	Prodynorphin
CALB2	5.36	Calbindin 2
GAD1	5.34	Glutamate Decarboxylase 1
CALB1	4.92	Calbindin 1
GSR	2.81	Glutathione-Disulfide Reductase
OPA1	2.01	OPA1 Mitochondrial Dynamin Like GTPase
CACNA1D	1.86	Calcium Voltage-Gated Channel Subunit Alpha1 D
NH (n = 13)		
BDNF-AS	17.38	BDNF Antisense RNA
BDNF	14.43	Brain Derived Neurotrophic Factor
GRIA3	12.44	Glutamate Ionotropic Receptor AMPA Type Subunit 3
P2RX2	9.84	Purinergic Receptor P2X 2
EMSLR	9.44	E2F1 MRNA Stabilizing LncRNA
PVALB	7.61	Parvalbumin
NTRK2	7.11	Neurotrophic Receptor Tyrosine Kinase 2
GPHN	5.94	Gephyrin
GSK3B	5.76	Glycogen Synthase Kinase 3 Beta
CABP4	4.20	Calcium Binding Protein 4
CACNA1D	1.92	Calcium Voltage-Gated Channel Subunit Alpha1 D
OPA1	1.63	OPA1 Mitochondrial Dynamin Like GTPase
SCN8A	1.00	Sodium Voltage-Gated Channel Alpha Subunit 8

^1^ Because the number of genes for the term “perception of sound” (PoS) in the GC data base was too low for comparison, further terms associated with normal hearing were included and summarized under the term “auditory perception” (AD; compare Figure 1 and Table 1). This list contains 25 unique genes.

**Table A2:** Genes identified by the keywords “cochlear nucleus” AND “synaptic transmission” AND “acoustic stimulation” (AcouStim, n = 30).

Gene	AcouStim Score	Name
BDNF-AS	18.2	BDNF Antisense RNA
BDNF	15.56	Brain Derived Neurotrophic Factor
GRIN1	10.3	Glutamate Ionotropic Receptor NMDA Type Subunit 1
P2RX2	9.95	Purinergic Receptor P2X 2
PVALB	9.67	Parvalbumin
GRM5	9.23	Glutamate Metabotropic Receptor 5
SYP	8.94	Synaptophysin
EMSLR	8.49	E2F1 MRNA Stabilizing LncRNA
GRM1	8.14	Glutamate Metabotropic Receptor 1
DRD1	7	Dopamine Receptor D1
MALAT1	6.89	Metastasis Associated Lung Adenocarcinoma Transcript 1
ADCY10	6.59	Adenylate Cyclase 10
NTRK2	6.55	Neurotrophic Receptor Tyrosine Kinase 2
GLRA1	6.38	Glycine Receptor Alpha 1
CALB1	6.31	Calbindin 1
SLC12A5	5.79	Solute Carrier Family 12 Member 5
CALB2	5.67	Calbindin 2
SLC17A6	5.66	Solute Carrier Family 17 Member 6
GAD1	5.48	Glutamate Decarboxylase 1
GLRB	5.13	Glycine Receptor Beta
FOS	4.95	Fos Proto-Oncogene, AP-1 Transcription Factor Subunit
FMR1	4.85	Fragile X Messenger Ribonucleoprotein 1
CREB1	4.65	CAMP Responsive Element Binding Protein 1
MAPK1	4.51	Mitogen-Activated Protein Kinase 1
GSR	2.74	Glutathione-Disulfide Reductase
EGR1	2.29	Early Growth Response 1
OPA1	2.29	OPA1 Mitochondrial Dynamin Like GTPase
BLZF1	2.03	Basic Leucine Zipper Nuclear Factor 1
CACNA1D	2	Calcium Voltage-Gated Channel Subunit Alpha1 D
JUN	1.56	Jun Proto-Oncogene, AP-1 Transcription Factor Subunit

**Table A3:** Genes identified by the keywords “tinnitus” AND “cochlear nucleus” AND “synaptic transmission” (n = 37).

Gene	Tinnitus Score	Name
BDNF-AS	21.22	BDNF Antisense RNA
BDNF	18.83	Brain Derived Neurotrophic Factor
GRIA2	15.41	Glutamate Ionotropic Receptor AMPA Type Subunit 2
P2RX2	11.36	Purinergic Receptor P2X 2
NTF3	10.85	Neurotrophin 3
GRIN2A	10.11	Glutamate Ionotropic Receptor NMDA Type Subunit 2A
SYP	9.93	Synaptophysin
GRIN1	9.49	Glutamate Ionotropic Receptor NMDA Type Subunit 1
MALAT1	8.93	Metastasis Associated Lung Adenocarcinoma Transcript 1
GRM5	8.91	Glutamate Metabotropic Receptor 5
EMSLR	8.69	E2F1 MRNA Stabilizing LncRNA
GRM1	8.31	Glutamate Metabotropic Receptor 1
PDYN	7.26	Prodynorphin
GPHN	6.55	Gephyrin
NGF	6.21	Nerve Growth Factor
CTNNB1	5.32	Catenin Beta 1
SLC17A6	5.27	Solute Carrier Family 17 Member 6
CALB2	5.15	Calbindin 2
GLRA1	5.01	Glycine Receptor Alpha 1
GAD1	4.94	Glutamate Decarboxylase 1
MAPK1	4.69	Mitogen-Activated Protein Kinase 1
GLRB	4.5	Glycine Receptor Beta
CACNA1D	4.49	Calcium Voltage-Gated Channel Subunit Alpha1 D
NTRK3	4.32	Neurotrophic Receptor Tyrosine Kinase 3
GSK3B	4.09	Glycogen Synthase Kinase 3 Beta
CREB1	4.02	CAMP Responsive Element Binding Protein 1
MAPK3	3.18	Mitogen-Activated Protein Kinase 3
FGF2	3.06	Fibroblast Growth Factor 2
BTD	3.05	Biotinidase
FOS	2.79	Fos Proto-Oncogene, AP-1 Transcription Factor Subunit
EGR1	2.64	Early Growth Response 1
NTRK1	2.58	Neurotrophic Receptor Tyrosine Kinase 1
ARSA	1.81	Arylsulfatase A
OPA1	1.46	OPA1 Mitochondrial Dynamin Like GTPase
JUN	1.33	Jun Proto-Oncogene, AP-1 Transcription Factor Subunit
SCN8A	1.31	Sodium Voltage-Gated Channel Alpha Subunit 8
PCAT1	0.95	Prostate Cancer Associated Transcript 1
